# New Adapted In Vitro Technology to Evaluate Biofilm Formation and Antibiotic Activity Using Live Imaging under Flow Conditions

**DOI:** 10.3390/diagnostics11101746

**Published:** 2021-09-23

**Authors:** Cassandra Pouget, Catherine Dunyach-Remy, Alix Pantel, Sophie Schuldiner, Albert Sotto, Jean-Philippe Lavigne

**Affiliations:** 1 Virulence Bactérienne et Infections Chroniques, INSERM U1047, Université de Montpellier, 30908 Nîmes, France; cassandra.pouget@gmail.com; 2 Virulence Bactérienne et Infections Chroniques, INSERM U1047, Université de Montpellier, Service de Microbiologie et Hygiène Hospitalière, CHU Nîmes, 30029 Nîmes, France; catherine.remy@chu-nimes.fr (C.D.-R.); alix.pantel@chu-nimes.fr (A.P.); 3 Virulence Bactérienne et Infections Chroniques, INSERM U1047, Université de Montpellier, Service des Maladies Métaboliques et Endocriniennes, CHU Nîmes, 30029 Nîmes, France; sophie.schuldiner@chu-nimes.fr; 4 Virulence Bactérienne et Infections Chroniques, INSERM U1047, Université de Montpellier, Service des Maladies Infectieuses et Tropicales, CHU Nîmes, 30029 Nîmes, France; albert.sotto@chu-nimes.fr

**Keywords:** antibiotics, biofilm, Bioflux^TM^ 200, chronic wounds, flow conditions, live imaging, living cells

## Abstract

The polymicrobial nature of biofilms and bacterial interactions inside chronic wounds are keys for the understanding of bacterial cooperation. The aim of this present study was to develop a technique to study and visualize biofilm in live imaging under flow conditions (Bioflux™ 200, Fluxion Biosciences). The Bioflux^TM^ system was adapted using an in vitro chronic wound-like medium (CWM) that mimics the environment encountered in ulcers. Two reference strains of *Staphylococcus aureus* (Newman) and *Pseudomonas aeruginosa* (PAO1) were injected in the Bioflux^TM^ during 24 h to 72 h in mono and coculture (ratio 1:1, bacteria added simultaneously) in the CWM vs. a control medium (BHI). The quantification of biofilm formation at each time was evaluated by inverted microscopy. After 72 h, different antibiotics (ceftazidime, imipenem, linezolid, oxacillin and vancomycin) at 1x MIC, 10x MIC and 100x MIC were administrated to the system after an automatic increase of the flow that mimicked a debridement of the wound surface. Biofilm studies highlighted that the two species, alone or associated, constituted a faster and thicker biofilm in the CWM compared to the BHI medium. The effect of antibiotics on mature or “debrided” biofilm indicated that some of the most clinically used antibiotic such as vancomycin or imipenem were not able to disrupt and reduce the biofilm biomass. The use of a life cell imaging with an in vitro CWM represents a promising tool to study bacterial biofilm and investigate microbial cooperation in a chronic wound context.

## 1. Introduction

Chronic wounds are a severe and expensive worldwide problem [1]. Wounds are considered chronic when the healing process fails to happen normally and the functional integrity of the skin is not achieved after more than 4 weeks [2]. Infections contribute to nonhealing wounds and are the consequence of microorganisms’ multiplication in the wound bed, leading to a prolonged excessive inflammatory response and delays in the re-epithelialization [3]. Chronic wounds include diabetic foot ulcers, pressure or decubitus ulcers and venous or arterial leg ulcers [4]. The cost of treatment, amputation, rehabilitation, and long-term care of these chronic wounds are estimated to be around USD 10.9 billion [1].

A biofilm is a sessile community of bacterial cells enclosed by a self-secreted extracellular polymeric matrix (EPS) present at the wound bed in 60–80% of cases [5,6]. These microbial communities have the potential to colonize and grow on surfaces of medical implants such as catheters or implants but also on tissues themselves [7]. Bacterial biofilms are a serious health problem due to their abilities to tolerate antibiotics, host defence systems and other external stresses contributing to the chronicity of the infection. Indeed, a biofilm EPS provides an additional resistance power of bacteria to tolerate stressful environments and antimicrobial agents, leading to the emergence of multidrug resistant bacteria [8]. Evidence showed that polymicrobial biofilm present in chronic wounds plays a major role in the inability of wounds to heal [9].

The chronic wound bed presents a complex microenvironment that typically includes more than one bacterial species interacting with each other [10]. Antibiotic therapy corresponds to one of the main treatments used to reduce the bacterial burden; however, there is a lack of evidence concerning its complete effectiveness in this clinical situation [11]. Despite this, patients with chronic wounds receive significantly more antibiotic prescriptions than other age- and gender-matched patients [12]. The physicians have to evaluate the role of each microorganism in the persistence of chronic wounds and distinguish between colonizing and infecting bacteria before applying antibiotic treatments [13]. The delicate balance between infection and colonization as well as the presence of sessile bacteria at the wound bed imply that most antibiotics are nonconclusive solutions to optimally treat chronic wound infections. Moreover, according to some publications, antimicrobial susceptibility depends on the environment in which bacteria evolved [14,15]. It is thus important to test the efficiency of antibiotics or new antibiofilm molecules in an environment closely related to the conditions encountered in vivo to clearly evaluate their potential and to avoid the risk of multidrug resistant bacteria emergence. The aim of this work was to describe a new in vitro model to study biofilm formation under flow condition in a medium mimicking chronic wounds environment. This model could be an interesting approach to a better knowledge on biofilm formation and organization but also to evaluate antimicrobial strategies in conditions close to the in vivo wound environment.

## 2. Materials and Methods

### 2.1. Bacterial Strains and Culture Conditions

All bacteria and media used in this study are listed in Table 1.

The composition of the in vitro chronic wound medium (CWM) (corresponding to European patent application EP21305337, filed on 18 March 2021) is presented in Table 1. To summarize, this new in vitro model is composed of a Bolton broth, a peptone-based medium of animal origin that makes it possible to model the nutrients likely to be present in the early stages following the debridement of the wound (e.g., damaged and degraded tissue and abundance of extracellular matrix compounds). Human serum and blood are also added to form the three major constituents found in the wound level, namely, red blood cells, serum and damaged tissues. Moreover, studying the chemical environment of the wound, we found that the pH is significantly increased to reach around 8. This basic pH is one of the main characteristics of a nonhealing wound caused by a modification due to the local oxygen deprivation and nutrient requirements. We consequently buffered our medium at pH 8.0 with 10 mM HEPES/NaOH. Finally, to mimic the cellular and inflammatory environment present in the wound, we added 1.10^6^/mL of human keratinocytes debris.

Bacteria were grown overnight in bacterial culture tubes under shaking at 200 rpm, in aerobic condition at 37 °C in the brain heart infusion (BHI, Sigma-Aldrich, Saint-Quentin-Fallavier, France) broth or in the CWM.

The antimicrobial susceptibility testing of isolates was performed by a broth microdilution method on the BHI medium according to EUCAST recommendations (https://www.eucast.org/clinical_breakpoints, accessed on 1 January 2021). The same procedure was applied in the CWM to compare the effect of this medium on antibiotics action.

### 2.2. Biofilm Formation of Monoculture and Mixed-Culture in a Microfluidic System

**Figure 1:**
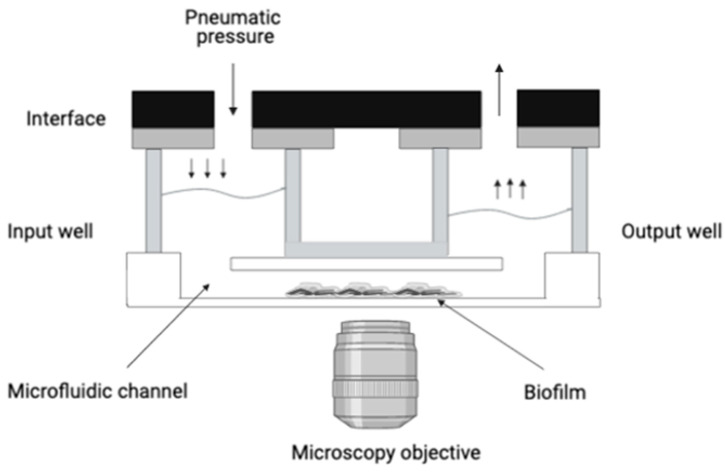
Schematization of the BioFlux^TM^ 200 organization. This system delivers controlled shear flow for simulating physiological and environmental conditions. It allows the connection by microchannel of an inflow well to an outflow well. Biofilm formation will occur in the microfluidic channel under shear flow condition. A microscope can be placed underneath the microfluidic channel, allowing a live observation of the biofilm formation.

To investigate biofilm formation, we used the microfluidic system of a BioFlux™ 200 (Fluxion Bioscience Inc., Alameda, CA, USA). The system included an inflow well connected by microchannel to an outflow well (Figure 1). Colonies of the strains were resuspended in 3 mL of BHI or CWM and were incubated at 37 °C with shaking (220 rpm) overnight. A bacterial suspension was then prepared from this overnight culture standardized to an OD_600_ of 0.1 ± 0.05 following two serial 1:200 dilutions. For the mixed culture, after the preparation of those bacterial suspensions, a mixed solution of *S. aureus* and *P. aeruginosa* was made. The primary step of the experiment consisted of priming the channel of the BioFlux^TM^ system with 500 µL of fresh medium in the inflow well with a pressure setting of 1 dyne/cm^2^ for 10 min. The remaining medium present in the well was then withdrawn. Thereafter, the microfluidic channels were inoculated by injecting the bacterial suspension of mono or mixed-culture from the output reservoir for 30 min at 1 dyne/cm^2^. The setup was placed on the heating plate at 37 °C. This step is important to enhance bacterial adhesion in the channel. Finally, the bacterial suspension was transferred in the inflow well for 72 h with pressure and temperature settings of 0.2 dyne/cm^2^ and 37 °C, respectively. Biofilms were obtained with bacteria cultivated either alone or in combination, in the BHI medium and in the CWM.

CFU counts on mixed biofilm (coculture of *S. aureus* Newman and *P. aeruginosa* PAO1) after 24 h, 48 h and 72 h inside the Bioflux ^TM^ 200 system in both the BHI and CWM media were performed by flow reversion in order to control the presence of each species. After dilutions, the bacteria were plated on nonselective (Luria–Bertani agar) and selective agar (cetrimide agar for *P. aeruginosa* and mannitol salt agar for *S. aureus*) plates. Three independent experiments were performed.

### 2.3. Gene Expression of P. aeruginosa Exopolysaccharides and Alginate Biosynthesis by Real-Time (RT)-PCR

Sessile bacteria were recovered from the microfluidic channel to the output well by an increase of the flow. We applied a pressure of 20 dynes/cm^2^ for 30 min. Bacteria were then pelleted by centrifugation and resuspended in 1 mL of PBS 1X. We analysed the mRNA (transcription) levels of the exopolysaccharides *pel*, *psl* and of the regulator of alginate biosynthesis *algD*. Total cellular RNA was extracted using the Rneasy^®^ Mini kit (Qiagen, Courtaboeuf, France) and treated with RNase-free DNase (Qiagen) for 30 min at 37 °C, after which a second step of purification was performed. RT-PCR was carried out in a LightCycler^®^ using the one-step LightCycler^®^ RNA Master SYBR Green I kit (Roche Applied Science, Meylan, France) according to the manufacturer’s protocol. The specificity of the generated products was tested by melting point analysis. Amplifications were performed in triplicate from three different RNA preparations. Cycle threshold (Ct) values of the target genes were compared with the Ct values of the housekeeping *rpoD* gene, chosen as an endogenous reference for normalizing the transcription levels of the target gene. The condition where bacteria were cultivated in a BHI medium was used as the control and the normalized relative expressions of the studied genes in the CWM were determined for each strain according to the equation 2−∆∆Ct, where ∆∆Ct = (Ct_gene_ − C_rpoD_) in the CWM and (Ct_gene_ − Ct_rpoD_) in the BHI medium. Primers used were listed in Table 2.

### 2.4. Automatized Debridement and Antibiotic Treatment in the Microfluidic System

In order to mimic a clinical debridement, which represents the first step of clinical management of a chronic wound, we developed a technique to remove part of the preformed biofilm. After 72 h of biofilm formation, a shear flow of 5 dynes/cm^2^ was applied from the input well to the output well for 10 min. The flow was then reversed from the output well for 10 min. This process of flow reversion was performed twice for debrided monomicrobial biofilm and 4 times for the debridement of a mixed polymicrobial biofilm. The aim of this flow inversion was to significantly reduce the biofilm constituted in the microfluidic channel.

After an analysis of clinical studies [2,20,21,22] concerning the debridement efficiency of the wound bed, we determined that the biofilm was never completely removed. There was always a percentage of remaining biofilm that facilitated its reformation. Clinical data estimated the effectiveness of debridement whether mechanical, surgical, enzymatic or other to be around 85% [23]. Thus, using flow inversion on the BioFlux^TM^ system, we tried to recreate this phenomenon of remaining preformed biofilm. Therefore, we fixed a maximum of 13 to 15% of remaining biofilm in the channel to mimic the in vivo conditions. The aim of this mechanical debridement was to further study the effect of antimicrobial molecules on preformed debrided biofilm. After the automatized debridement, a dynamic flow (0.2 dyne/cm^2^ at 37 °C) of antibiotics was applied to evaluate their actions on the remaining preformed biofilm for 24 h. All antibiotics and the concentrations used in this study are listed in Table 3.

To confirm the effect of antibiotics, a determination of dead bacteria among the disrupted biofilm was performed as previously published [24]. Briefly, bacteria released from the biofilm were collected in the output well after 24 h of antibiotics exposure in the BioFlux^TM^ 200 in both the BHI medium and the CWM. The total number of bacteria present in the suspension was calculated by measuring the optical density at 600 nm and concentrations were obtained following our standard curve. After dilutions, bacteria were plated on nonselective (Luria–Bertani agar for monocultures) or selective agar plates (cetrimide agar for *P. aeruginosa* and mannitol salt agar for *S. aureus* for coculture). CFU counts were then performed giving the number of living bacteria present in the output well. The number of dead bacteria was calculated by the difference between the total number of bacteria and the number of living bacteria. Finally, the percentage of dead bacteria corresponded to the ratio: number of dead bacteria/total number of bacteria. Three independent experiments were performed.

### 2.5. Visualisation and Quantification of Biofilm

After 24 h, 48 h and 72 h of incubation, biofilm formation was recorded using a fluorescence inverted microscope DM IRB (Leica Biosystems, Nanterre, France) coupled with a CoolSNAP FX camera (Roper Scientific, Lisses, France). A video was recorded in real time at a rate of 60 frames per seconds. MetaVue^TM^ software (Molecular Devices, Sunnyvale, CA, USA) was used for imaging. ImageJ^®^ was utilized to colour black and white images, to include scale bars and to calculate biofilm percentage. The 16-bit grayscale images were adjusted with the threshold function to fit the bacterial structure and were analysed using the “Analyze Particles” function. The percentage of biofilm was evaluated before and after automatized debridement and after 24 h of antibiotics treatment following the same protocol as previously described [25,26].

### 2.6. Statistical Analysis

Statistical analyses were performed using GraphPad Prism version 7 or R version 3.5.2. Tests used for the *p* value determination are mentioned in each figure legend.

## 3. Results

### 3.1. Evaluation of Biofilm Formation in the Bioflux^TM^ System

To evaluate the potential of biofilm formation under BioFlux^TM^ flowthrough conditions of two of the main species isolated from chronic wounds, we determined the percentage of biofilm formation of *S. aureus* Newman, *P. aeruginosa* PAO1 and a mixed culture in the control BHI medium and the new CWM (better adapted to mimic the environment encountered in wounds) developed in our unit.

**Figure 2:**
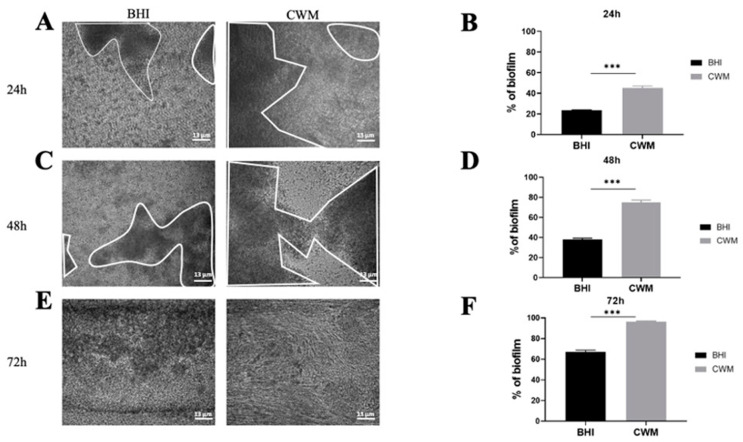
Kinetics of the biofilm formation of *Staphylococcus aureus* Newman in the BioFlux^TM^ system. (**A**,**C**,**E**) correspond to representative images taken at different times ((**A**) 24 h; (**C**) 48 h and (**E**) 72 h) of biofilm formation in the CWM and BHI media. The images are the result of one of the three experimental triplicates. Biofilm is visualized by the black part in between the white lines. Percentages of biofilm formation are presented at 24 h (**B**), 48 h (**D**) and 72 h (**F**) postincubation in both media after three independent experiments. They were determined by the ImageJ software application. Results are presented as the mean ± standard deviation. Statistics were performed using a *t*-test on GraphPad Prism version 7. Ns, *p* > 0.05; *** *p* < 0.001.

**Figure 3:**
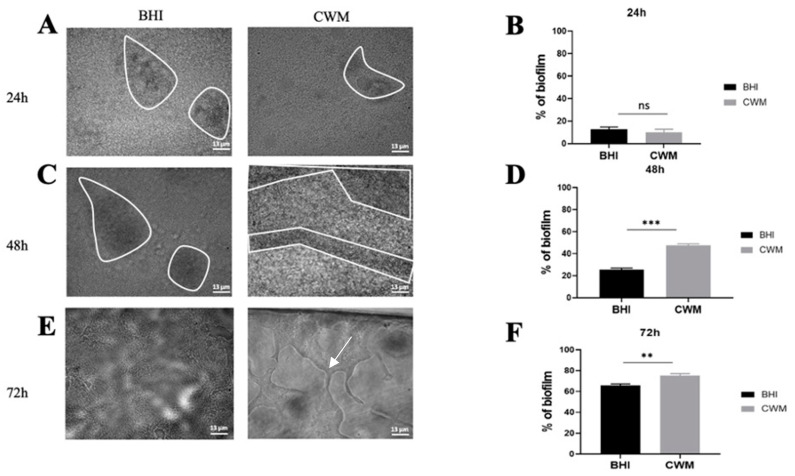
Kinetics of biofilm formation of *Pseudomonas aeruginosa* PAO1 in the BioFlux^TM^ system. (**A**,**C**,**E**) correspond to representative images taken at different times ((**A**) 24 h; (**C**) 48 h and (**E**) 72 h) of biofilm formation in the CWM and BHI media. The images are the result of one of the three experimental triplicates. Biofilm is visualized by the black part in between the white lines. Percentages of biofilm formation are presented at 24 h (**B**), 48 h (**D**) and 72 h (**F**) postincubation in both media after three independent experiments. They were determined by the ImageJ software application. The white arrow indicates the presence of the exopolysaccharides structures formed by *P. aeruginosa* during biofilm formation. Results are presented as the mean ± standard deviation. Statistics were performed using a *t*-test on GraphPad Prism version 7. Ns, *p* > 0.05; ** *p* < 0.01; *** *p* < 0.001.

The two reference strains were able to form biofilm and to remain attached under shear force (Figure 2 and Figure 3).

The *S. aureus* Newman strain formed a biofilm at each time point (Figure 2). Interestingly, at 24 h the percentage of constituted biofilms ranged from 22.0% ± 0.2 when the strain was cultivated in the BHI medium to 46.5% ± 0.4 in the CWM (*p* < 0.001) (Figure 2B). This significant difference was increased at 48 h post inoculation with 36.0% ± 0.2 of bacteria in the biofilm within the BHI medium vs. 77.2% ± 0.5 within the CWM (*p* < 0.001) (Figure 2D). Finally, after 72 h, the percentage of formed biofilm in CWM was almost complete (98.3% ± 0.1), whereas it was still incomplete in the BHI medium (63.1% ± 0.3) (*p* < 0.001) (Figure 2F). The significant difference in the biofilm formation of *S. aureus* in the CWM compared to the BHI medium are visualized in Figure 2A,C,E.

**Figure 4:**
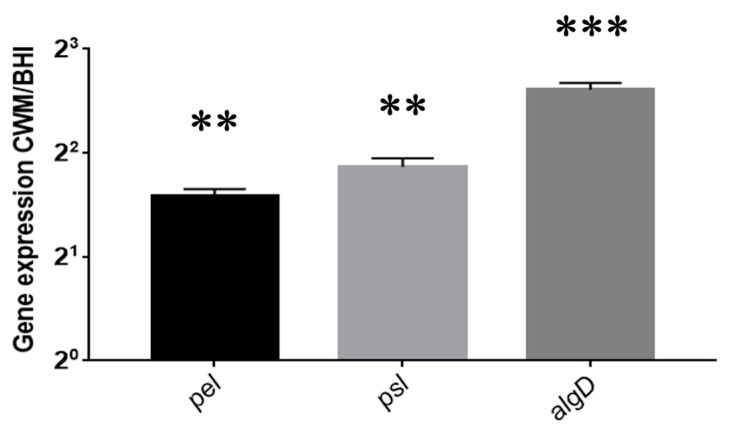
Expression of *Pseudomonas aeruginosa* PAO1 *pel*, *psl* and *algD* genes after recovering sessile cells in the BioFlux^TM^ system after 72 h of culture. The normalized relative expressions of the studied genes in the CWM were determined for each strain according to the equation 2−∆∆Ct, where ∆∆Ct = (Ct_gene_ − C_rpoD_) in the CWM-(Ct_gene_ − Ct_rpoD_) in the BHI medium. Results are presented as the mean ± standard deviation of three different experiments. Statistics were performed using a *t*-test on GraphPad Prism version 7. ** *p* < 0.01; *** *p* < 0.001.

*P. aeruginosa* biofilm formation inside the Bioflux^TM^ system is shown in Figure 3. Contrary to *S. aureus*, no difference in the percentage of biofilm formation has been observed after 24 h post inoculation in the CWM (14.1% ± 0.4) vs. BHI medium (16.0% ± 0.3) (*p* = Not Significant (NS)) (Figure 3B). However, significantly higher percentages of biofilm constituted in the CWM compared to the BHI medium were noted at 48 h (49.4% ± 0.1 and 22.0% ± 0.1, respectively) (Figure 3D) and at 72 h (75.7% ± 0.2 and 63.1% ± 0.1, respectively) (*p* < 0.001) (Figure 3F). The coloured pixels in the images (Figure 3A,C,E) confirmed the presence of a more intense biofilm in the CWM after 48 h. Interestingly, the presence of particular structures has been observed at 72 h in the CWM (Figure 3E). To understand those particular structures (arrow in Figure 3E) observed after 72 h of monoculture of *P. aeruginosa* cultured in the CWM in the Bioflux^TM^ system, the gene expressions of the exopolysaccharides, *pel* and *psl*, as well as the regulator of alginate biosynthesis, *algD*, were analysed by qRT-PCR. Those three extracellular polysaccharides have been implicated in biofilm development. The results are shown in Figure 4. A significant difference in the expression of the exopolysaccharides-encoding genes, *pel* (*p* < 0.01) and *psl* (*p* < 0.01), was observed after 72 h of culture. Moreover, the expression of a key regulator of the alginate production, *algD*, was also significantly increased in the CWM compared to the BHI (*p* < 0.001).

**Figure 5:**
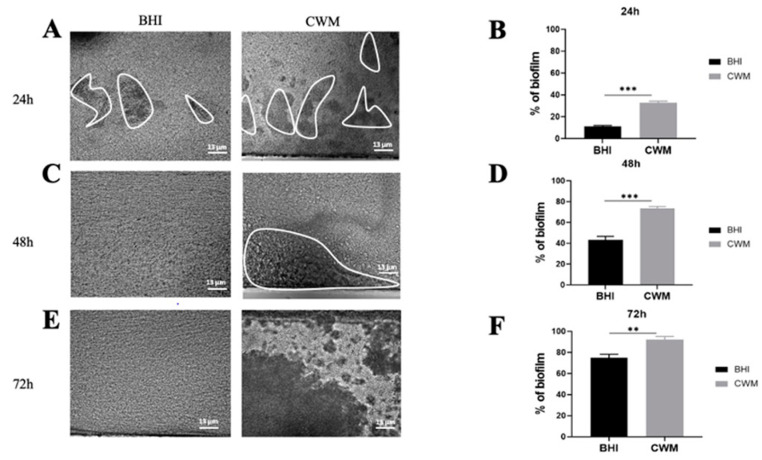
Kinetics of biofilm formation of coculture of *Staphylococcus aureus* Newman and *Pseudomonas aeruginosa* PAO1 in the BioFlux^TM^ system. The ratio of coculture used was 1:1. The two species were added into the system simultaneously. (**A**,**C**,**E**) correspond to representative images taken at different times ((**A**) 24 h; (**C**) 48 h and (**E**) 72 h) of biofilm formation in the CWM and BHI media. The images are the result of one of the three experimental triplicates. Biofilm is visualized by the black part in between the white lines. Percentages of biofilm formation are presented at 24 h (**B**), 48 h (**D**) and 72 h (**F**) postincubation in both media after three independent experiments They were determined by the ImageJ software application. Results are presented as the mean ± standard deviation. Statistics were performed using a *t*-test on GraphPad Prism version 7. Ns, *p* > 0.05; ** *p* < 0.01; *** *p* < 0.001.

Finally, inside a mixed biofilm composed of *S. aureus* and *P. aeruginosa*, the two species formed a faster and denser biofilm in the CWM compared to the BHI medium at any time point (Figure 5). Indeed, a significant difference in the percentage of biofilm formation was noted at 24 h (36.0% ± 0.3 in the CWM vs. 17.4% ± 0.2 in the BHI medium) (Figure 5B), at 48 h (78.0% ± 0.2 and 41.0% ± 0.1, respectively) (Figure 5D) and at 72 h (93.3% ± 0.2 and 72.0% ± 0.4, respectively) (Figure 5F). It is also interesting to note that *S. aureus* formed a biofilm more rapidly than *P. aeruginosa* PAO1 at 24 h (*p* < 0.001) and at 48 h (*p* < 0.01) in the CWM. Moreover, in coculture, *P. aeruginosa* PAO1 established a more important biofilm in coculture compared to monoculture in the CWM (*p* < 0.001) and the BHI medium (*p* < 0.1). To confirm that the mixed biofilm was equally composed by *S. aureus* and *P. aeruginosa*, CFU counts were performed inside the BioFlux^TM^ 200. No statistical difference in the proportion of bacteria isolated in the BHI medium and the CWM at the same times was observed (Table S1).

### 3.2. Antibiotics Activity on Preformed Biofilm Is Dependent on the Culture Media

To mimic the management of chronic wounds, we adapted an automatized debridement of the biofilm inside the BioFlux^TM^ system and administrated different antibiotics on preformed biofilms by *S.* *aureus* Newman, *P. aeruginosa* PAO1 and mixed culture in the BHI medium and the CWM.

The MIC values for each antibiotic against the studied strains are shown in Table 2.

**Figure 6:**
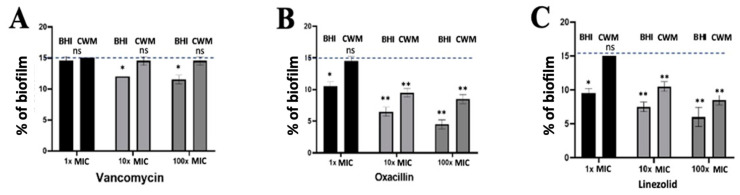
Percentage of biofilm reduction on preformed *Staphylococcus aureus* Newman biofilm in the BioFlux^TM^ system using vancomycin (**A**), oxacillin (**B**) and linezolid (**C**) at different concentrations (1x, 10x and 100x MIC) and in the CWM and BHI media. An automatized debridement was fixed at 15% of biofilm left in the microfluidic channel. Samples were tested in three independent experiments. Results are presented as the mean ± standard deviation. Statistics were performed using a *t*-test on GraphPad Prism version 7. Ns, *p* > 0.05; * *p* < 0.1; ** *p* < 0.01.

After the constitution of a biofilm with *S. aureus* Newman, no effect on the reduction of this preformed biofilm was observed using 1x MIC of vancomycin regardless of the medium used (14.7% ± 0.3 in the BHI medium and 15.0% ± 0.1 in the CWM) (*p* = NS) (Figure 6A). At 10 x and 100x MIC of vancomycin, a significant reduction of the preformed biofilm was shown in the BHI medium (11.9% ± 0.2) (*p* < 0.1), whereas no effect was noted when the biofilm was constituted in the CWM (14.8% ± 0.1) (*p* = NS) (Figure 6A). No difference in the percentage of dead bacteria was noted on disrupted biofilm regardless of the concentration of vancomycin used (Table S2). Using oxacillin, a significant reduction in the percentage of preformed biofilm was observed at 1x MIC in the BHI medium (10.8% ± 0.2) (*p* < 0.1) (Figure 5B). Interestingly, an important reduction of the biofilm was detected using 10 and 100x MIC of oxacillin in both media (in the BHI medium: 7.4% ± 0.3 and 4.7% ± 0.4, respectively; in the CWM: 8.9% ± 0.3 and 8.2% ± 0.3, respectively) (*p* < 0.01) (Figure 6B). This observation was correlated with a significant increase in the percentage of dead bacteria detected in the two media (*p* < 0.01) (Table S2). The same observation was noted using linezolid. A reduction of the preformed biofilm at 1x MIC was only shown in the BHI medium (9.8% ± 0.3) (*p* < 0.1), whereas the decreased percentage of biofilm was more important at 10 and 100x MIC, regardless of the medium used (in the BHI medium: 7.2% ± 0.2 and 6.7% ± 0.3, respectively; in the CWM: 10.6% ± 0.3 and 8.5% ± 0.2, respectively) (*p* < 0.01) (Figure 6C). We could note that regardless of the antibiotic used and their concentrations, their action on preformed biofilm was significantly reduced in the CWM compared to the BHI medium (*p* < 0.01).

**Figure 7:**
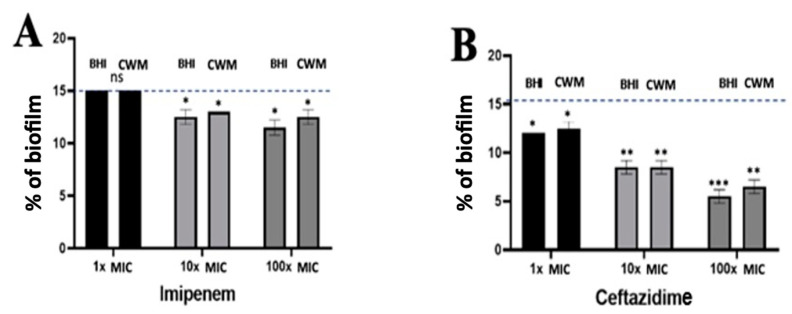
Percentage of biofilm reduction on preformed *Pseudomonas aeruginosa* PAO1 biofilm in the BioFlux^TM^ system using imipenem (**A**), and ceftazidime (**B**) at different concentrations (1x, 10x and 100x MIC) and in the CWM and BHI media. An automatized debridement was fixed at 15% of biofilm left in the microfluidic channel. Samples were tested in three independent experiments. Results are presented as the mean ± standard deviation. Statistics were performed using a *t*-test on GraphPad Prism version 7. Ns, *p* > 0.05; * *p* < 0.1; ** *p* < 0.01; *** *p* < 0.001. Antibiotics efficiency on preformed *Pseudomonas aeruginosa* PAO1 biofilm.

Using *P. aeruginosa* PAO1, no effect could be noted on the reduction of the preformed biofilm when 1x MIC of imipenem was administrated regardless of the medium used (15.0% ± 0.1 in the BHI medium and 14.9% ± 0.2 in the CWM) (*p* = NS) (Figure 7A). When the antibiotic concentration was increased (10x MIC and 100x MIC), a significant reduction of the biofilm could be observed with similar results in both media (in the BHI medium: 13.4% ± 0.2 and 12.3% ± 0.2, respectively; in the CWM: 13.7% ± 0.4 and 13% ± 0.3, respectively) (*p* < 0.01) (Figure 7A). Interestingly, 1x MIC of ceftazidime had a significant impact on the preformed biofilm (12.4% ± 0.2 in the BHI medium and 12.9% ± 0.4 in the CWM) (*p* < 0.1). This trend was increased at 10x MIC (7.6% ± 0.2 in the BHI medium and 7.7% ± 0.3 in the CWM) and 100x MIC (5.6% ± 0.3 in the BHI medium and 6.4% ± 0.4 in the CWM) regardless of the medium used (*p* < 0.01) (Figure 7B). We could note that both media had no influence on the antimicrobial action of ceftazidime and imipenem against *P. aeruginosa.* The percentage of dead bacteria showed significant results at 10x and 100x MIC of ceftazidime (*p* < 0.01) (Table S2).

**Figure 8:**
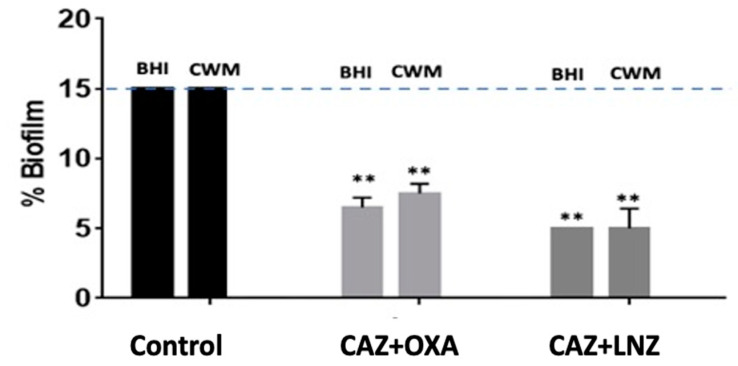
Percentage of biofilm reduction on preformed *Staphylococcus aureus* Newman and *Pseudomonas aeruginosa* PAO1 mixed biofilm in the BioFlux^TM^ system using 10x MIC of ceftazidime (CAZ) + oxacillin (OXA) and 10x MIC of ceftazidime (CAZ) + linezolid (LNZ) in the CWM and BHI media. An automatized debridement was fixed at 15% of biofilm left in the microfluidic channel. Samples were tested in three independent experiments. Results are presented as the mean ± standard deviation. Statistics were performed using a *t*-test on GraphPad Prism version 7. Ns, *p* > 0.05; ** *p* < 0.01.

Finally, when an optimized combination of antibiotics (10x MIC of ceftazidime + 10x MIC of oxacillin and 10x MIC of ceftazidime + 10x MIC of linezolid) was used on a preformed biofilm constituted by *S. aureus* Newman and *P. aeruginosa* PAO1, a significant reduction of the preformed mixed biofilm was observed in both media when the association of ceftazidime and oxacillin (6.7% ± 0.2 in the BHI medium and 7.1% ± 0.3 in the CWM) and of ceftazidime and linezolid (5.6% ± 0.1 in the BHI medium and 6.2% ±0.7 in the CWM) were used (*p* < 0.01) (Figure 8). The efficiency of the combination of antibiotics was significantly increased in comparison to the antibiotic used alone on a single species at the same concentration (*p* < 0.01). This observation was correlated with a significant increase in the percentage of dead bacteria detected when we used these combinations (*p* < 0.01) (Table S2). As observed with *P. aeruginosa* alone, the two media had no influence on the antimicrobial action of the combination of antibiotics.

## 4. Discussion

Biofilm formation is a crucial step in the pathophysiology of chronic wounds [6]. Its study is of concern and requires the development of new tools [27]. Classically, biofilms are investigated using in vitro experiments in static conditions (e.g., crystal violet method, resazurin, plates count, or microscopy). These traditional microbiological techniques have provided great knowledge about bacterial biofilm potential. However, they present several limitations, such as difficulties in culturing some bacteria, the study of polymicrobial biofilm, the heterogeneity of bacterial populations inside a biofilm or difficulties in recreating some physical or biological conditions [28]. Microfluidics technologies are new and emerging technologies that complement current biological assays. Their evaluation is important to estimate their potential. In this study, we showed that the BioFlux^TM^ system was a particularly adapted and interesting strategy to study dynamic biofilm formation in mono or mixed culture, to mimic the debridement (a crucial step in the eviction of the biofilm, notably in wounds) and to characterize the effect of antimicrobial agents against a preformed and debrided mono or polymicrobial biofilm. Moreover, it has been clearly established that the behaviour of microorganisms was highly dependent on the environment in which they evolved [6]. The environment encountered by bacteria in clinical situations influences bacterial virulence and its potential for biofilm formation. The development of a new in vitro medium mimicking in vivo conditions remains an important challenge to evaluate the bacterial behaviour in a specific environment and the effect of therapeutic solutions. Therefore, our group has adapted the “Chronic Wound-like Medium” with the aim to be closer to physiological environments encountered in wounds.

Inside the BioFlux^TM^ system, we observed that if the two studied species formed a robust biofilm after 72 h as expected, species cultivated in the CWM had a significant higher ability to facilitate microbial adhesion and its subsequent biofilm formation compared to the traditional microbiological medium (BHI) (*p* < 0.001). The *S. aureus* Newman strain was particularly influenced by the CWM and formed a faster and denser biofilm compared to *P. aeruginosa* PAO1. Differences in the biofilm formation of the species might underline that the culture medium had an important impact on the bacterial behaviour as previously observed [29]. This reinforces the need to cultivate bacteria in an environment that closely mimics the in vivo conditions. Bacterial culture media have different compositions that can influence bacterial phenotype such as fitness, early adhesion, biofilm formation or genes expression [30]. To corroborate these constatations, we observed a different behaviour of a same species in the two studied media in our study. Alginate is an exopolysaccharide synthesized to respond to environmental conditions. It is produced by *P. aeruginosa* as the most abundant extracellular matrix polysaccharide and regulated virulence, biofilm formation and resistance to different antibiotics [31]. It has been previously shown that the reference strain of *P. aeruginosa* PAO1 was a non-mucoid strain, which does not exhibit any alginate production in the conventional culture media [32]. However, in our study, we observed the presence and production of structures harbouring the characteristics of exopolysaccharides as alginate, when this strain was cultivated for 72 h in the CWM (Figure 3E) contrary to the BHI medium. After extended cultivation (72 h in our study), we observed that *P. aeruginosa* PAO1 strain up-regulated expression of the gene involved in biosynthesis of the alginate but also of exopolysaccharides-encoding genes (*pel* and *psl*). Those three components are keys for creating a strong scaffold involved in maintaining biofilm integrity. Those data suggested that in the CWM mimicking stressful conditions, bacteria could produce a stronger and denser biofilm compared to a classical microbiological culture medium. It also proves that this new in vitro medium promotes a strong and noticeable scaffold of exopolysaccharide. *algG* is involved in the alginate biosynthetic operon that is required for the incorporation of L-guluronate residues into alginate. So far, observations corroborated the fact that the strains had different behaviour depending on the media used for culture. We could also note that a medium mimicking wound conditions improved biofilm formation by influencing the secretion of proteins involved in the biofilm process (e.g., alginate). The surfaces used to study biofilm formation also represent an important parameter that influences microbial adhesion. In static models, polystyrene is the main component of the adherent surfaces used controversially for dynamic assays where glass is predominant. Surface-dependent attachment is especially more pronounced in in vitro assays where no human matrix proteins such as fibrinogen or fibronectin, which normally serve as anchors for attachment, are present [33]. The microfluidic model used in this study allows precoating on the glass support of the shear model damaged human cells, such as keratinocytes or melanocytes, to mimic the cellular environment present at the wound bed, which serves as adhesion support [34].

The investigation of biofilms must also include the action of antimicrobial agents. Biofilms are involved in more than 80% of chronic wounds infections [35]. The penetration of antibiotics is usually reduced in biofilm due, among others, to the EPS matrix [7]. Thus, the antimicrobial effect of antibiotics is compromised within these environments. Biofilms of *P. aeruginosa* and *S. aureus* are particularly relevant in chronic wounds, since they are most of the time isolated together at the wound bed and their interactions create an additional difficulty to eradicate the biofilm [36]. Two types of in vitro biofilm models are currently used to predict antimicrobial efficiency against biofilm: closed and open systems. Closed systems are characterised by limited nutrients and accumulation of metabolic waste which can create a bias in biofilm quantification [37]. Open systems better reproduce the conditions encountered in vivo, as there is a permanent control of nutrient delivery, flow, and temperature [38]. The BioFlux^TM^ system is a microfluidic open system in which multiple biofilms can be run at the same time [39] and where protocols adaptation to mimic in vivo biofilm formation or biofilm management is possible (e.g., automatized debridement for chronic wounds). In this study, we evaluated different antibiotics commonly used against *S. aureus* and *P. aeruginosa* infections. They serve as a model, acting at different levels in bacteria and presenting variable activities against biofilm formation. In the BioFlux^TM^ system, we were able to test antibiotics alone or in combination, at different concentrations and in different media on mono or polymicrobial biofilms. The multiplicity of studies available with this technology is reinforced by the possibility to obtain image intensity results, which can be translated to a remaining biofilm percentage evaluating the efficiency of antibiotics on a preformed biofilm. Thus, we highlighted a variety of antibiotics action in our study. Oxacillin, linezolid and ceftazidime were particularly active alone or in combination against a preformed biofilm whereas vancomycin and imipenem had clearly few activities. Even at 100x MIC, vancomycin was unable to reduce the *S. aureus* preformed biofilm. This difference could be explained by the ability of linezolid or β-lactams to penetrate deeper into the biofilms and to be able to directly come into contact with the bacteria. Interestingly, although the two antibiotics (imipenem and ceftazidime) used against *P. aeruginosa* were not influenced by the culture medium tested, a significant difference could be observed with the antibiotics used against *S. aureus*. These antibiotics presented a lower ability to decrease the monomicrobial preformed biofilm cultured in the CWM compared to the BHI medium. This observation is of concern and could explain the difficulties to treat common microbial-biofilm-associated infections where bacteria are installed in this chronic situation even if the antibiotic seems active against the bacteria following the antibiogram results. Regarding the recent literature data on *P. aeruginosa* biofilm and the efficacy of antibiotics, several studies have highlighted an inefficiency of the imipenem at sub-MIC and MIC concentrations to reduce bacteriological biomass within the biofilm. Authors have shown that the use of imipenem even favoured the maturation and increase of bacterial biomass in biofilm from clinical isolates of cystic fibrosis [40,41]. Only Musafer et al. [42] demonstrated an action of imipenem on three clinical strains isolated in a context of cystic fibrosis. However, those conclusions were drawn with a static in vitro model of biofilm formation. Concerning the ceftazidime effect, some studies concur with the conclusions presented in this article. Otani et al. [43] noted the effectiveness of ceftazidime to reduce the PAO1 biofilm even at sub-MIC concentrations. The efficacy of ceftazidime was also demonstrated in an in vivo model of ureteral stent infection in combination with another antibiotic (azithromycin) [44]. This antibiotic therefore has a very valuable antibiofilm potential but could be modulated. Indeed, it was reported by several studies that despite the important antibiofilm potential of ceftazidime, the long-term use of the antibiotic can cause an increase in resistance in the planktonic and sessile population [45,46]. For *S. aureus* strains, the literature on the effect of vancomycin as a potential antibiofilm is consistent with what this study highlighted. Vancomycin at MIC concentration are not capable of generating a reduction in bacterial density within the biofilm [47]. At sub-MIC concentrations, vancomycin use increased the formation of mature biofilm [48]. Some studies highlighted an effect on sessile bacteria; however, the concentrations used were particularly high (90–120x MIC) or the administration time was too long to be clinically useful [49,50]. There are few data on the use of oxacillin on biofilm during the early stage of formation or on preformed mature biofilm. Mirani et al. [51] demonstrated an influence of this antibiotic on *S. aureus* reference strains. The mechanism could be due to the modulation of the *icaA* and *agr* expression, two major regulator genes of biofilm formation. The efficacy of oxacillin on mature biofilm was confirmed by Manner et al. [52]. Finally, regarding linezolid, publications reported that this antibiotic used alone was ineffective to reduce the biofilm of *S. aureus* [53] notably in osteoarticular infections [54]. Only Gander et al. [55] observed an effect of 1x MIC on the early stage of biofilm formation but with a classic microbiological medium and in static conditions. Those data thus reinforce the need to use more reliable and standardized media and models to evaluate the antimicrobial agents and their potential effects and risks. This also validates the interest in the BioFlux^TM^ system in these investigations.

## 5. Conclusions

*S. aureus* and *P. aeruginosa* biofilms are implicated in several infections. In chronic wounds, pathological biofilm is frequent and difficult to treat. Thus, new therapeutic strategies are needed and the inhibition of preformed biofilms is a great challenge for the treatment of these infections. Therefore, in vitro biofilm models should be improved in clinical microbiology to predict the efficiency of antimicrobial treatments on sessile cells. The combination of a new chronic wound medium and the BioFlux^TM^ microfluidic system represents a powerful tool to study biofilm formation and to screen the potential antibiofilm actions of candidate molecules under conditions mimicking those encountered in vivo.

## Figures and Tables

**Table 1:** Bacterial strains and media used in the study.

**Strain**	**Characteristics**	**References**
PAO1	*Pseudomonas aeruginosa* reference strain	[16]
Newman	*Staphylococcus aureus* reference strain	[17]
**Media**	**Characteristics**	**References**
BHI	Brain Heart Infusion, Reference medium for bacterial culture	Sigma-Aldrich
CWM	Chronic wound medium, medium mimicking in vivo conditions encountered in chronic wounds. Composition: 79.5% Bolton broth, 20% decomplemented human serum, 0.5% haemolysed human blood, HaCaT debris	European patent application EP21305337

**Table 2:** Primers used in the study.

Primers Used and Target Function	Target Region	Primer Name	Oligonucleotide Sequence	Reference
GDP-mannose 6-dehydrogenase	*algD*	algD-F algD-R	5′-CGTCAACGTCAACGTCGTG-3′ 5′-AACAGCAGCTTGCCCTTGTA-3′	[18]
Exopolysaccharides	*pel*	pel-F pel-R	5′-AGCAAGAAAGGAATCGCCG-3′ 5′-GACCGACAGATAGGCGAAGG-3′	[19]
Exopolysaccharides	*psl*	psl-F psl-R	5′-CTGCCCTCACCTTTCGCC-3′ 5′-GGAAGGATCAGCTGCG-3′	[19]
Housekeeping gene	*rpoD*	rpoD-F rpoD-R	5′-CGATCGGTGACGACGAAGAT-3′ 5′-GTTCATGTCGATGCCGAAGC-3′	[18]

**Table 3:** Antibiotics used and minimum inhibitory concentration determined in the study.

Antibiotics	Strains	Concentrations Used in the Preformed Biofilm	EUCAST MIC Breakpoints (mg/L)	MIC in the BHI Medium (mg/L)	MIC in the CWM (mg/L)
Vancomycin (Mylan)	*S. aureus* Newman	1, 10, 100x MIC	2	1	1
Oxacillin (Astellas)		1, 10, 100x MIC	ND ^1^	0.5	1
Linezolid (Fresenius Kabi)		1, 10, 100x MIC	4	1	1
Imipenem	*P. aeruginosa* PAO1	1, 10, 100x MIC	4	1	2
Ceftazidime (PanPharma)		1, 10, 100x MIC	8	1	1

^1^ ND, not defined.

## Data Availability

Data supporting reported results can be found on the BioFlux software in our Unit at Bioflux experiments—PAO1 and Newman—Kinetics and ATB effect.

## Supplementary Materials

The following are available online at https://www.mdpi.com/article/10.3390/diagnostics11101746/s1, Table S1: Percentage of *S. aureus* Newman and *P. aeruginosa* PAO1 present inside the mixed biofilm formed in the Bioflux ^TM^ 200 system after 24 h, 48 h and 72 h in both the BHI medium and the CWM and obtained by flow reversion. Results are expressed as the mean ± standard deviation of three independent experiments. Statistics were performed using a t-test on GraphPad Prism version 7. Ns, non-significant. Table S2: Percentage of dead bacteria of *Staphylococcus aureus* Newman (A) and *Pseudomonas aeruginosa* PAO1 (B) alone or combined in a mixed biofilm (C) in the BioFlux^TM^ 200 system after exposition to antibiotics in the different media. Samples were tested in three independent experiments. Results are presented as the mean ± standard deviation. Statistics were performed using a t-test on GraphPad Prism version 7. Ns, non-significant.
