# Supplementary material for: New Adapted In Vitro Technology to Evaluate Biofilm Formation and Antibiotic Activity Using Live Imaging under Flow Conditions
Source: Diagnostics (Basel). 2021 Sep 23;11(10):1746. doi: 10.3390/diagnostics11101746 (PMC8535051; doi:10.3390/diagnostics11101746)
Supplement: Supplementary file 1 [file diagnostics-11-01746-s001.zip › diagnostics-1336736-supplementary.pdf]

Supplementary material

Table S1. Percentage of *S. aureus* Newman and *P. aeruginosa* PAO1 present inside the mixed biofilm formed in the Bioflux ™ 200 system after 24-hr, 48-hr and 72-hr in both BHI and CWM and obtained by flow reversion. Results are expressed as the mean ± standard deviation of three independent experiments. Statistics were performed using a t-test on GraphPad Prism version 7. ns, non-significant.

	***P. aeruginosa* PAO1**		***S. aureus* Newman**		***p (PAO1 vs Newman)***	
	**BHI**	**CWM**	**BHI**	**CWM**	**BHI**	**CWM**
**24-hr**	45% ± 0.5%	44% ± 0.7%	55% ± 0.9%	56% ± 0.4%	ns	ns
**48-hr**	48% ± 0.8%	47% ± 0.5%	52% ± 0.7%	53% ± 0.6%	ns	ns
**72-hr**	48% ± 0.7%	46% ± 0.5%	52% ± 0.9%	54% ± 0.7%	ns	ns

Table S2. Percentage of dead bacteria of *Staphylococcus aureus* Newman (A) and *Pseudomonas aeruginosa* PAO1 (B) alone or associated in a mixed biofilm (C) in the BioFlux™ system after the exposition to antibiotics in the different media. Samples were tested in three independent experiments. Results are presented as the mean ± standard deviation. Statistics were performed using a t-test on GraphPad Prism version 7. ns, non-significant.

A.

S. aureus **Newman**	**% of dead bacteria**		**P With vs without ATB**	
	BHI	CWM	BHI	CWM
**Without ATB**	5% ± 0.5	6% ± 0.6	-	-
**1x MIC Oxacillin**	7% ± 0.4	5% ± 0.4	ns	ns
**10x MIC Oxacillin**	20% ± 0.8	19% ± 0.5	<0.01	<0.01
**100x MIC Oxacillin**	24% ± 0.9	18% ± 0.6	<0.01	<0.01
**1x MIC Linezolid**	12% ± 0.9	8% ± 0.7	ns	ns
**10x MIC Linezolid**	19% ± 0.7	16% ± 0.8	<0.01	<0.01
**100x MIC Linezolid**	22% ± 0.8	20% ± 0.9	<0.01	<0.01
**1x MIC Vancomycin**	6% ± 0.8	6% ± 0.8	ns	ns
**10x MIC Vancomycin**	11% ± 0.8	6% ± 0.7	ns	ns
**100x MIC Vancomycin**	13% ± 0.6	7% ± 0.8	ns	ns

B.

P. aeruginosa **PAO1**	**% of dead bacteria**		**P With vs without ATB**	
	BHI	CWM	BHI	CWM
**Without ATB**	5% ± 0.8	6% ± 0.9	-	-
**1x MIC Imipenem**	5% ± 0.8	6% ± 0.8	ns	ns
**10x MIC Imipenem**	10% ± 0.9	8% ± 0.8	ns	ns
**100x MIC Imipenem**	13% ± 0.8	12% ± 0.7	ns	ns
**1x MIC Ceftazidime**	14% ± 0.7	13% ± 0.9	ns	ns
**10x MIC Ceftazidime**	19% ± 0.6	16% ± 0.7	<0.01	<0.01
**100x MIC Ceftazidime**	26% ± 0.5	23% ± 0.8	<0.01	<0.01

C.

P. aeruginosa **PAO1 +** ***S. aureus*** **Newman**	**% of dead bacteria**		**P With vs without ATB**	
	BHI	CWM	BHI	CWM
**Without ATB**	5% ± 0.8	6% ± 0.7	-	-
**10x MIC Oxacillin+ceftazidime**	22% ± 0.9	20% ± 0.8	<0.01	<0.01
**10x MIC Linezolide + ceftazidime**	30% ± 0.8	28% ± 0.8	<0.01	<0.01
